# Compassionate Extracorporeal Membrane Oxygenation Discontinuation: A Narrative Review and Practical Process Model for Reliable End-of-Life Care

**DOI:** 10.3390/healthcare14091249

**Published:** 2026-05-06

**Authors:** Kinsley Hubel, Raju Reddy, Akram Khan, Jonathan Pak, Nehan Sher

**Affiliations:** 1 Division of Pulmonary, Allergy & Critical Care Medicine, Oregon Health & Science University, 3181 SW Sam Jackson Park Road, UHN-67, Portland, OR 97239, USA; hubel@ohsu.edu (K.H.); pakj@ohsu.edu (J.P.); 2 Division of Pulmonary and Critical Care Medicine, University of Texas at Austin, Austin, TX 78712, USA; raju.reddy@austin.utexas.edu; 3 Division of Pulmonary, Critical Care & Sleep Medicine, University of Arizona College of Medicine, Phoenix, AZ 85004, USA; sher.neha@gmail.com

**Keywords:** extracorporeal membrane oxygenation, palliative care, withholding treatment, intensive care units, end-of-life care

## Abstract

Background and Objectives: Extracorporeal membrane oxygenation (ECMO) provides temporary respiratory or circulatory support when conventional therapies fail. Some patients do not recover and are not candidates for transplant or durable mechanical support. In these cases, continuing ECMO may no longer align with the patient’s goals. Compassionate ECMO discontinuation (CED) is the planned withdrawal of extracorporeal support with death anticipated. The term “compassionate” refers to the goal of minimizing suffering in the end-of-life process. This review proposes a reliability-oriented framework to standardize CED and reduce preventable distress for patients, families, and clinicians. Materials and Methods: We conducted a targeted narrative review of ethical analyses, consensus guidance, and empirical literature on planned ECMO withdrawal. The results of the narrative review were combined with our existing practical process for CED into this proposed reliability-oriented framework as a guide for clinicians. Recommendations were organized into a four-phase process model that emphasizes operational implementation, anticipatory guidance, and quality improvement. We included modality-specific considerations for veno-arterial (VA), veno-venous (VV) ECMO, and extracorporeal cardiopulmonary resuscitation (ECPR). Results: The framework includes four phases. Phase I, Anticipation and Alignment, emphasizes structured shared decision-making, early expectation setting, time-limited trials, palliative care integration, and predefined pathways for managing disagreement. Phase II, Preparation, includes interdisciplinary role assignment, a pre-withdrawal time out, family coaching on expected physiological changes, and preemptive comfort medications that account for ECMO-altered pharmacokinetics. Phase III, Implementation, prioritizes comfort first, pacing with explicit pause points, environmental controls to reduce alarms and visual distress, and modality-tailored sequencing. Phase VI, Aftercare and Learning Capture, includes bereavement support, standardized documentation, structured team debriefing, and recommended process measures to guide improvement. Conclusions: Viewing CED as a low-frequency, high-stakes clinical process supported by scripts, checklists, and iterative feedback can improve goal-concordant end-of-life (EOL) care, reduce suffering and family trauma, support clinicians, and strengthen ECMO program learning systems.

## 1. Introduction

Extracorporeal membrane oxygenation (ECMO) provides temporary gas exchange and/or circulatory support for patients with potentially reversible organ failure or as a bridge to transplant or durable device therapy. The two most common modalities of ECMO are veno-venous (VV) ECMO and veno-arterial (VA) ECMO. ECMO utilization has expanded substantially over the past two decades, with the Extracorporeal Life Support Organization (ELSO) registry documenting over 154,000 runs globally between 2009 and 2022 across 57 countries, and with recent years seeing ~10,000 or more ECMO runs per year reported internationally [1]. Withdrawal of life-sustaining therapy accounts for a substantial proportion of ECMO-associated deaths, reported in 53–76% of cases across cohorts [2,3]. Even in high-performing centers, a substantial proportion of patients do not recover sufficiently to warrant ongoing support. In a contemporary cohort, 40.1% of patients supported with VA ECMO were not successfully weaned [4]. These patients, surrogates, and clinicians then face a transition from life-extending therapy to end-of-life (EOL) care, in which discontinuation of ECMO is planned with death expected.

Qualitative studies of life support withdrawal in intensive care more broadly suggest that family involvement in withdrawal of life-sustaining treatment is frequently experienced as emotionally overwhelming and distressing, with some families describing the process as traumatic [5]. Trust in the treating physician plays a critical role, as families are less likely to agree to withdrawal when trust in the clinician team is lacking [6]. With ECMO, this transition requires discontinuing a highly visible, invasive technology in real time at the bedside. This process can amplify distress when communication, symptom control, and technical implementation are not tightly coordinated.

Currently, there is a gap in the available literature focusing on the discontinuation of ECMO with expected death. This lack of guidance leaves institutions and care teams to develop their own process for facilitating this care transition. Transitioning off ECMO in the end-of-life process is a high-risk, low-frequency event, and the lack of a standardized process reduces the likelihood that the transition goes smoothly for the patient, family, and clinical team. A reliability-oriented framework focuses on eliminating catastrophic failures to achieve zero harm in a high-hazard environment. The focus is on early detection of potential problems, anticipating risks, and improving staff engagement. This review uses a reliability-oriented framework to reduce the potential failure to alleviate symptoms and thereby the distress experienced by the patient, family, and care team. A more traditional framework places the focus on ethical principles and consensus rather than the learning mindset approach of the reliability-oriented framework.

Compassionate ECMO discontinuation (CED) has been used to describe the planned withdrawal of ECMO when ongoing support no longer aligns with an achievable, patient-centered goal (e.g., recovery, transplant, durable mechanical support) and death is expected [7,8]. CED aims to minimize suffering and align care with patient values, but it is also a technically complex, emotionally charged care transition for patients, families, and staff. While CED is often described as a discrete ethical decision (“whether and when to withdraw”) rather than a complex, high-stakes intensive care unit (ICU) procedure, harm can occur even when the decision to compassionately discontinue ECMO is ethically sound. In practice, variability in preparation, communication, medication timing, and technical execution can produce predictable failure modes: delayed symptom control, conflicting messages to families, distressing alarms and blood exposure, or abrupt physiologic collapse without anticipatory guidance, resulting in patient distress, family trauma, and moral distress among clinicians [7,8,9,10]. Accordingly, this proposed process model reframes CED as a high-risk care transition that requires reliability, defined roles, and anticipatory planning [11,12,13]. To operationalize this reframing, we synthesize actionable recommendations and provide practical tools to support implementation, including our proposed domain-based checklist that aligns with our four-phase reliability-oriented framework, an electronic medical record documentation template, a shared decision-making framework, example conversational scripts to help clinicians explain the clinical and technical aspects of ECMO withdrawal to families, and example institutional protocols. Together, these resources aim to reduce process variability, improve communication, and minimize preventable distress (Online Supplement, Appendix A, Appendix B, Appendix C, Appendix D and Appendix E). This review focuses on adult ECMO programs and provides modality-specific recommendations for VA and VV ECMO. The modality-specific recommendations will allow ECMO centers to anticipate the potential challenges that come with weaning each modality and provide a framework for expectation setting with patients and families about the CED process.

## 2. Methods

### 2.1. Design and Reporting Approach

We conducted a structured narrative review of clinical guidance, ethical analyses, professional society guidance statements, and selected empirical literature addressing planned withdrawal of extracorporeal support with death anticipated. Given the heterogeneity of available evidence, including empirical studies, consensus statements, and normative ethical analyses, a narrative approach was selected to enable a practice-oriented synthesis rather than quantitative meta-analysis. We combined the findings of this review with expert-informed institutional practice to build the proposed reliability-oriented process model for CED.

### 2.2. Objectives

We sought to characterize how CED is described across empirical studies, consensus statements, and ethics analyses, with emphasis on bedside processes and reliability. Specifically, we aimed to identify (1) clinical contexts and triggers that prompt consideration of CED; (2) communication practices and shared decision-making approaches for patients and surrogates; (3) comfort-focused clinical and technical practices, including VA versus VV ECMO considerations and concurrent devices; and (4) post-event practices, including bereavement support, debriefing, and clinician moral distress.

### 2.3. Literature Identification and Information Sources

A structured search of MEDLINE (via PubMed) was performed from database inception through 19 October 2025. The search strategy used a combination of keywords and Medical Subject Headings (MeSH), including “Extracorporeal Membrane Oxygenation, Withholding Treatment, Palliative Care, Terminal Care, Life Support Care, and Ethics, Medical” using Boolean operators. The complete PubMed search string is provided in Supplementary Material(S1). This search yielded 305 articles published between 1989 and 2025. Additionally, a targeted review of publications relevant to ECMO decision-making and withdrawal practices from the Extracorporeal Life Support Organization (ELSO), American Thoracic Society, American Medical Association, and Society of Critical Care Medicine was identified, along with reference lists from key guidance documents and foundational articles.

### 2.4. Selection and Inclusion Approach

Eligible articles were published in English, and they addressed ethical frameworks, consensus recommendations, institutional guidance, and empirical data related to decision-making about CED in adult patient populations. Sources were selected iteratively based on conceptual relevance to planned withdrawal of ECMO with death anticipated and applicability to adult ICU practice. We prioritized documents offering actionable recommendations for communication, symptom management, and ECMO-specific operational or technical processes. Pediatric or neonatal-only sources were used selectively when they provided generalizable communication or process insights. Where multiple sources addressed similar domains, preference was given to recent consensus statements and studies with explicit descriptions of bedside practices. Study selection was intended to support rigorous narrative synthesis rather than an exhaustive capture of all available literature. Of the 305 MEDLINE articles found using the search and a further 5 publications from professional society guidance, we incorporated a total of 60 articles in this review. A classification framework mapping evidence type to recommendations is provided in the Supplementary Table S1.

### 2.5. Synthesis of Domains

We organized the narrative synthesis around recurring, practice-relevant domains: clinical context and patient population; ECMO modality (VA, VV, ECPR) when described; triggers and decision points; ethical and legal considerations; communication and documentation practices; symptom-management approaches; technical aspects of discontinuation (including VA/VV differences and adjunct devices); and post-event practices (bereavement, debriefing, staff support). Domains were iteratively refined as new sources were identified and mapped to the emerging reliability-oriented process framework.

### 2.6. Assessment of Evidence Base

Because the review integrates heterogeneous source types (empirical studies, consensus guidance, and normative analyses), we did not conduct a formal risk-of-bias assessment. Instead, we classified the evidentiary basis of recommendations according to whether they were supported primarily by empirical data, expert consensus, or ethical argumentation. We also note when evidence was indirect (e.g., extrapolated from general ICU withdrawal literature) versus ECMO-specific. There is rare empirical evidence available regarding CED, and what is available focuses predominantly on pharmacokinetics and pharmacodynamics of medication use in ECMO.

### 2.7. Framework Development

We developed a reliability-oriented framework that conceptualizes compassionate ECMO discontinuation as a high-risk clinical process requiring anticipatory risk identification, deliberate preparation, coordinated execution, and structured follow-through. Recommendations were organized to reduce predictable sources of harm, including misalignment among teams, unanticipated symptom burden, technical or sequencing errors, family distress, and clinician moral injury. Ethical analysis was incorporated by mapping recommendations to established biomedical ethics principles and ICU approaches to managing potentially inappropriate treatments, with emphasis on operationalizing these principles at the bedside. In addition to the literature synthesis, our institutional CED process, refined through iterative clinical use over the past two years, was integrated into the final framework. The resulting model reflects both published evidence and practice-based refinement and is intended as an adaptable structure for diverse institutional contexts.

## 3. Four-Phase Reliability-Oriented Process Model

This review focuses on the operational conduct of planned ECMO withdrawals as a comfort-focused bedside process. Using thematic synthesis across domains, we organized recommendations into a four-phase, reliability-oriented process model that reflects the temporal and operational sequence of CED. Implementation tools (scripts, documentation template, and sample protocols) are provided in the Online Supplement (Appendix A, Appendix B, Appendix C, Appendix D and Appendix E). There exists very little empirical data to guide end-of-life care in CED. However, there are consensus statements and literature supporting how to navigate end-of-life care in the general ICU populations. The consensus statements, extrapolation of literature from general ICU population, and expert opinion have formed the basis for our four-phase reliability-oriented process model. We would like to acknowledge the limitations in applicability, as each institution may use different equipment, staffing models, and policies related to end-of-life care and ECMO management. Furthermore, the legal permissibility of CED will vary significantly by country, state, and institution. We recommend obtaining ethics and legal consultation at your institution to determine the feasibility of CED. Our recommendations are intended to complement current ELSO guidance and are designed to be used alongside ELSO’s living guideline set for patient care, VV ECMO, VA ECMO, ECPR, and patient transport [14].

*Checklist for Compassionate ECMO Discontinuation (CED)*

A comprehensive checklist that maps the domains to the four phases of our model is in Table 1. This checklist is intended to reduce preventable harm during CED by promoting shared situational awareness, anticipatory symptom management, and coordinated interdisciplinary execution *during* a high-risk, low-frequency event. It is not intended to replace individualized clinical judgment or ethical deliberation. Used as intended, the checklist functions as a pre-procedure brief: it surfaces latent safety threats (missing medications, unclear roles, unresolved family questions) while there is still time to correct them, and it creates a shared script that helps the team move through the four phases of our reliability model [15,16].

### 3.1. Phase I—Anticipation and Alignment

Phase I emphasizes early alignment, shared mental models, explicit goals, and anticipatory guidance as foundational “reliability work” before the bedside CED sequence begins. Phase I clarifies why CED should be approached as a high-risk clinical process and not solely as a discrete “withdrawal decision.” The recommendations in Phase I are extrapolated from the literature about general ICU EOL care, combined with expert opinion regarding ECMO-specific considerations during EOL care.

#### 3.1.1. Terminology

We use “CED” to refer to planned discontinuation of ECMO when death is anticipated, following a goals-of-care decision that ongoing extracorporeal support no longer aligns with an achievable, patient-centered outcome. CED is distinct from (1) elective decannulation after recovery; (2) discontinuation for transition to another destination therapy (e.g., transplant or durable mechanical support); and (3) unplanned cessation due to circuit failure or emergent complications.

#### 3.1.2. Modality-Specific Scope

We distinguish VA ECMO from VV ECMO because the expected physiology and tempo after discontinuation differ materially, and therefore require different anticipatory guidance and technical sequencing. In VA ECMO, cessation commonly precipitates rapid circulatory collapse and may occur alongside other mechanical circulatory support devices. In VV ECMO, patients more often experience progressive hypoxemia and hypercarbia over minutes to hours as sweep gas is reduced. Accordingly, subsequent sections specify modality-tailored approaches to pacing, adjunct device management, and symptom-focused ventilator changes. Extracorporeal Cardiopulmonary Resuscitation (ECPR) is a subset of VA ECMO but with specified candidacy criteria that can necessitate early reassessment and time-limited trials compared to patients placed on VA ECMO outside of ECPR. ECPR programs should integrate early alignment and contingency planning for possible CED during the consent and post-cannulation discussions with family or surrogate decision makers (SDM) [17].

#### 3.1.3. Decision-Making Scope

When patients have decision-making capacity, withdrawal planning should occur through shared decision-making and (when feasible) be structured to support meaningful family interaction without compromising comfort. When patients lack such capacity, clinicians should communicate clearly with SDMs about what to expect, particularly regarding cessation of ECMO support, ventilator changes, and extubation, to align the plan with the patient’s values and goals. Structured goals-of-care conversation frameworks (e.g., REMAP: Reframe, Expect emotion, Map out goals, Align, Propose a plan) can help standardize this communication choreography and reduce mixed messaging during high-stakes ECMO decisions (Online Supplement, Appendix A) [18,19,20].

#### 3.1.4. Ethical Framing

CED is frequently framed as an ethical decision of “whether and when to withdraw” care. However, the bedside harms that patients, families, and clinicians experience often arise from process variability rather than from the decision itself. CED is a low-frequency, high-stakes ICU event that produces predictable failure modes that are preventable. These failure modes result from inconsistent preparation, poor communication, medication timing issues, and bedside implementation, and they result in patient distress, family trauma, and moral distress among clinicians [2,3,4,5]. Phase I treats alignment work (shared mental models, time-limited goals, role clarity, and anticipatory guidance) as the first reliability safeguard for CED. This reliability framing also clarifies why ethical and legal considerations must be operationalized early, before crisis-driven decisions or inconsistent bedside practices amplify harm. Due to legal variability across countries and states, we recommend early legal consultation to determine if CED is permissible. As a pragmatic reliability tool, we recommend a consistent, teachable goals-of-care framework (such as REMAP) to structure reframing, emotional acknowledgment, values elicitation, alignment, and recommendation [18,19,20].

#### 3.1.5. Bridge to Phase II

In preparation for transitioning to Phase II, it is important to ensure that interdisciplinary consensus is obtained and documented, palliative care is involved, and goals of care have been documented. It is also important that a shared understanding of how unexpected ECMO circuit failure prior to CED initiation would be managed. After completing these core Phase I elements, the team is ready to begin Phase II—Preparation.

### 3.2. Phase II—Preparation

Phase II focuses on translating alignment into an executable plan, informed by consensus guidance, pharmacologic principles, extrapolation from ICU end-of-life care literature, and our practice-informed expertise. Elements included should be adapted to local institutional policies and workflows.

#### 3.2.1. Technical Considerations

CED requires attention to several clinical factors. Patients on ECMO may require higher-than-usual doses of sedatives and analgesics due to circuit-related pharmacokinetic changes, medication tolerance from prolonged support, and rapid physiologic shifts during discontinuation [21,22]. Ex vivo studies on the pharmacokinetics and pharmacodynamics of sedatives and analgesics demonstrate that medications with high protein binding (greater than 70%) and high lipophilicity (logP greater than 2) are highly susceptible to circuit sequestration [22]. However, the increased clearance or sequestration of these medications seems to plateau after 6–24 h and has minimal ongoing impact on dosing [23,24,25]. The available literature suggests significant dosing adjustments based on circuit-related changes will be minimal for any sedative or analgesic medication the patient has been on for more than one day; however, if initiating new medications for comfort at the time of CED, the patient may require higher-than-expected doses [23,24,25]. Hydromorphone has low lipophilicity and protein binding, and is likely to require fewer morphine milligram equivalents compared to fentanyl [26]. For VAECMO in particular, comfort medications should be administered before flow reduction to ensure adequate systemic distribution [7,27]. Clinicians should also anticipate that extubation may precipitate acute pulmonary edema on VA ECMO or abrupt dyspnea and increased work of breathing on VV ECMO, especially in patients previously requiring high ventilator support. Given the complexity of these decisions, early involvement of palliative care can support anticipatory planning and provide guidance for families and care teams [27,28]. At the end of life, discontinuation of VA and VV ECMO produces distinct physiologic trajectories. VA ECMO cessation typically results in rapid circulatory collapse, often with decreased consciousness due to low systemic perfusion, though agitation may occur if residual cardiac function persists. In contrast, VV ECMO withdrawal leads to progressive hypoxemia and hypercarbia, with dyspnea and air hunger as prominent terminal symptoms [7,27]. The rate of decline is determined by underlying ventilatory dependence. Extubation is reasonable during VA ECMO or VV ECMO withdrawal once comfort is achieved on minimal ventilatory support with adequate secretion control, acknowledging the risk of abrupt pulmonary edema in VA ECMO. These differences guide the withdrawal strategy, with VA ECMO emphasizing blood flow reduction to zero with circuit clamping and VV ECMO focusing on sweep gas flow reduction to zero to limit circuit manipulation. Phase II translates this physiologic understanding into an operational plan: the room is prepared, roles are assigned (Online Supplement, Appendix D), medications are prepared and administered proactively, and the team agrees on explicit “pause points” to reassess comfort and family readiness before the first irreversible step is taken [7,15,16]. In other words, preparation ends only when the team can reliably implement a comfort-first sequence without improvisation.

#### 3.2.2. Components

It is important to review the CED plan with the patient and family and ensure that any important rituals or legacy needs have been met. Once the family has a clear understanding of the CED process, the focus should shift to team preparation. We recommend that the team perform a Pre-Withdrawal Time Out, similar to a standard procedural time out. The time out should include a review of the planned sequence of withdrawal, including ventilator management, adjunctive therapy removal, mechanical circulatory support removal (VA ECMO only), and ECMO weaning and discontinuation with anticipated physiologic changes (Table 1). Anticipated symptoms should be assessed, and medications to manage them prepared. If the patient has been on neuromuscular blockade infusion, this should be stopped, and confirmation that the patient is no longer experiencing the effects must be obtained. Adjunct comfort measures such as music, family presence, antiemetics for nausea, and anti-cholinergics for secretion management should also be considered and implemented.

#### 3.2.3. Bridge to Phase III

Once these Phase II elements are completed, medications initiated, roles clarified, environment prepared, and family briefed, the team is positioned to implement CED as a coordinated bedside procedure. Phase III begins with a comfort checkpoint and proceeds in deliberate steps, with repeated reassessment to ensure that physiology does not outpace symptom control.

### 3.3. Phase III—Implementation

Phase III is the procedural moment of CED: a time-limited, leader-directed sequence that prioritizes comfort, minimizes chaos, and maintains a consistent narrative for the family. Implementation should be paced to the patient’s symptom trajectory rather than to device mechanics alone, and when distress appears, the sequence should be paused, comfort re-established, and only then can the team proceed [15,29]. To reduce role confusion, a single clinician should be designated to (i) lead the sequence, (ii) call explicit pause points, and (iii) maintain closed-loop communication with the ECMO specialist/perfusionist, bedside nurse, and respiratory therapist [7,27,29]. A second clinician (often palliative care or the primary intensivist) can focus on family-facing communication, explaining what is happening in plain language, preparing them for visible changes, and reinforcing that comfort is the clinical priority. Suggested bedside language and roles for these moments are provided in the Online Supplement (Appendix B and Appendix D). We provide an example of our institution’s device-specific VV and VA-specific protocols in the Online Supplement as an illustrative example of an exemplar protocol (Appendix E). These would need to be adapted using local institutional policies and reviewed with the respective institution’s specialists.

#### 3.3.1. Technical Considerations

The patient room should be prepared to serve as a calm environment, and this should include reducing alarms and removing non-essential equipment to maximize space for patient and family interactions. Once the team, family, and patient are ready, begin the transition off adjunctive therapies. There is no data to support a specific sequence of therapy removal. However, we recommend the following sequence to maximize patient time with family prior to ECMO cessation:

- Continuous renal replacement therapy (CRRT) cessation. This also allows for additional device removal from the room.
- Extubation, if desired by patient and family. Depending on the clinical situation, this may facilitate patient-family conversations.
- Deactivating ICD or pacemakers.
- Discontinuing vasopressor and inotrope infusions.
- Weaning and deactivating mechanical circulatory support devices such as ventricular assist devices or balloon pumps.

If extubation is desired, it should occur only after a period of stable comfort on minimal ventilatory support, with clear anticipatory guidance that breathing patterns may change substantially after extubation, even when suffering is well-controlled [7,27].

#### 3.3.2. Comfort Checkpoints

At minimum, the team should reassess comfort (facial expression, ventilator synchrony, respiratory pattern/effort, agitation, and family-observed distress) (i) before any reduction in ECMO support, (ii) after each major step change (sweep gas reduction or blood flow reduction), and (iii) immediately before extubation if planned [15,29]. These checkpoints create predictable opportunities to titrate medications proactively rather than reactively.

#### 3.3.3. ECMO-Specific Discontinuation

There are many ways to transition off ECMO, and there is no evidence favoring one approach over another. Our practice-driven recommendations are based on our institutional expertise and have been further honed through our own quality improvement processes. This serves as a guide that can be adapted to local devices and protocols. Regardless of ECMO modality, we recommend turning off alarms if feasible on the respective ECMO device and setting low-blood-flow alarms to 0 L/min. This is essential in VA ECMO and important even in VV ECMO to prevent alarms during and after the patient’s death. Alarm ringing during the process disrupts the calm environment and can result in significant patient, family, and staff distress.

VV ECMO: We recommend focusing on reducing the sweep gas flow to zero, effectively “clamping” the circuit without the need for cessation of blood flow. This has minimal intrusion into the patient-family interactions and allows for seamless weaning of ECMO support. Maintain the device FiO_2_ (FdO_2_) at 100%. The sweep gas should be weaned slowly enough to allow for titration of comfort medications. The speed of sweep gas weaning often depends on the patient’s initial level of required support. Because dyspnea and air hunger are dominant risks as sweep gas approaches zero, VV ECMO withdrawal should be framed to the family as a gradual transition in which comfort is reassessed repeatedly. When distress emerges, the team should treat symptoms first (opioid/benzodiazepine titration; secretion control) and only then resume sweep reduction.

VA ECMO: VA ECMO discontinuation often has a faster physiological tempo; therefore, medication administration and confirmation of comfort should precede the first decrement in blood flow. Families should be prepared for rapid changes in perfusion and responsiveness, and clinicians should explicitly state that visible changes do not imply suffering when comfort medications are effective. VA ECMO withdrawal may occur alongside other mechanical circulatory support devices. The leader should state the planned device sequence out loud (what will be turned down first, what will be turned off, and what changes the family may see) to prevent “silent steps” that may feel abrupt or confusing [7,27]. Maintain the device FiO_2_ (FdO_2_) at 100% and sweep gas flow at the current level. We recommend focusing on reducing pump speed or blood flow to the lowest tolerated by the respective ECMO device, ensuring medication administration before each wean in blood flow to allow for medication circulation. Then clamp the circuit (arterial return limb first, followed by venous drainage limb) close to the device to reduce intrusion into the patient-family space. Once the circuit is clamped, power down the device to prevent inadvertent alarms. We recommend covering the circuit and tubing to reduce any visual distress for the family as the blood separates within the tubing.

#### 3.3.4. Bridge to Phase IV

After ECMO support is discontinued, clinicians should remain present and attentive. Comfort reassessment continues until death occurs, and the family’s experience is shaped by what happens in these final minutes—tone, pacing, explanations, privacy, and visible commitment to non-abandonment. Phase IV begins immediately after death and extends beyond the bedside, focusing on documentation, bereavement support, team debriefing, and system learning so that future CED events are safer and less morally injurious.

### 3.4. Phase IV—Aftercare and Learning Capture

Documentation and bereavement support begin immediately after death, with deliberate attention to privacy, clarity, and closure for the family and the care team. Because CED is both a clinical procedure and a highly emotional ICU event, Phase IV serves two functions: (i) it reduces the risk of complicated grief and clinician moral residue through structured aftercare, and (ii) it converts an inherently low-frequency event into an opportunity for reliability improvement through learning capture [30,31].

#### 3.4.1. Documentation

Documentation should be clear and consistently use neutral, non-stigmatizing language (e.g., “compassionate ECMO discontinuation with death expected”). We recommend documenting CED as a procedure in the medical record. Documentation should include the sequence of withdrawal, symptom management strategies, and family presence. A sample ICU procedure note template is provided in the Online Supplement, Appendix C. Documentation should also support improvement by capturing operational details that are otherwise quickly lost (who led the sequence, whether medications were available without delay, whether alarms/noise/blood visibility were managed as planned, and whether any unanticipated distress occurred and how it was treated). These details enable teams to distinguish “expected physiology” from “process defects,” which is essential for building a learning system around a rare event.

#### 3.4.2. Aftercare and Support

After death, the team should offer unhurried time at the bedside, normalize a range of emotional responses, and support requested rituals or cultural practices whenever feasible [30,31]. Clear communication about next steps (pronouncement, device removal timing if applicable, what will happen to lines/tubes, and how the body will be cared for) reduces uncertainty and prevents families from feeling rushed or excluded. When feasible, continuity matters: having a known clinician return to the room to answer questions can be experienced as a final act of care.

A brief, structured debrief should be considered part of Phase IV, not optional “extra work.” The goal is to identify what went well, what created friction, and what should change next time—especially around symptom control timing, role clarity, environment management, and family communication [32,33]. Where patterns emerge (e.g., recurrent medication delays, unclear leadership, inadequate room setup, or inconsistent scripts), teams should route these observations into unit- or program-level quality improvement and checklist refinement. Over time, this feedback loop is what converts CED from a variable, high-risk event into a teachable, repeatable clinical process [32,33,34].

## 4. Ethical and Legal Aspects

Ethical challenges in ECMO care include appropriate patient selection, stewardship of this resource-intensive therapy in the setting of limited benefit, and the development of “bridge-to-nowhere” situations (situations where ECMO no longer serves as a bridge to recovery/transplant/destination therapy, yet the patient can physiologically persist on ECMO—creating prolonged ICU dependence with no path to discharge) as well as the ethical distinction between withholding and withdrawing ECMO [35]. Although clinicians and families may feel a greater emotional burden when stopping a treatment already in place, ethically, there is no distinction between choosing not to start an intervention and deciding to withdraw it later. If continued treatment no longer serves the patient’s expressed aims for care, clinicians may ethically discontinue that therapy [36,37]. Phase I prioritizes early conversations to prevent patients from becoming “stranded” on ECMO without an achievable destination.

As with all other aspects of clinical care, attention to the core principles of biomedical ethics—respect for autonomy, beneficence, non-maleficence, and justice—should guide decision-making in CED [38]. CED often reflects tension among core ethical principles, as respect for autonomy supports honoring the preferences of capacitated patients, while bridge-to-nowhere scenarios raise concerns of non-maleficence and justice related to prolonged suffering and resource use; however, these concerns do not override autonomy in patients with decisional capacity [10,35]. Although clinicians may determine that continued ECMO is harmful, and certain jurisdictions even allow legal protections to withdraw life-sustaining treatment over objection [39], unilateral withdrawal in an alert patient who does not agree to it raises concerns under non-maleficence because of the potential for profound emotional harm. Consistent institutional withdrawal processes promote justice by ensuring procedural fairness and, in practice, provide a shared script and pathway that reduces inconsistency across teams and shifts [40].

Schou et al. describe an ethics quick framework for ECMO: (i) anticipate the “bridge-to-nowhere” problem before cannulation, discuss this with the family prior to initiating ECMO support, and obtain an early palliative care consult; (ii) balance professional integrity and patient autonomy when disagreement arises; and (iii) recognize that ECMO may heighten common ICU ethical tensions such as high cost, limited evidence, and scarce alternatives, so utilize structured shared decision-making and time-limited trials [41]. Operationally, this framework maps directly onto Phase I tasks: prevent stranded trajectories, define decision points, and normalize time-limited trials as part of ECMO initiation and continuation.

Physicians have an obligation to limit non-beneficial ECMO care when the patient is determined to have no reasonable expectation of survival outside the ICU setting [42]. It is recommended that the ECMO team establishes negotiated, time-limited trials with the patient and their family, and engages the local institutional conflict-resolution pathways if needed [43,44]. Observational data show that the withdrawal of ECMO is common and context-dependent (e.g., noncandidacy for transplant, severe neurologic injury). The ECMO team should clarify that a “no-escalation” strategy is ethically legitimate when a patient becomes stranded on a “bridge-to-nowhere”, and pair this with explicit re-evaluation points for staff, patient, and family [2,41]. These points should be stated in plain language, documented, and revisited at a predictable cadence to reduce drift and mixed messaging.

At times, shared decision-making may not be achievable despite repeated meetings and clear prognostic communication. This can result in the family or patient wanting to continue ECMO despite the medical team’s assessment that ongoing ECMO support would be medically non-beneficial. This has the potential to result in the medical team’s pursuit of unilateral CED over a capacitated patient’s objection. If this situation arises, consensus among the ECMO faculty should be achieved, ethics and legal consultation should be obtained, and local institutional policies should be adhered to. Framing these steps as a predefined institutional pathway (rather than an ad hoc escalation) supports procedural fairness and reduces moral distress for bedside clinicians.

ECPR raises distinct consent and selection issues due to the emergency context and equipoise. ECMO programs should discuss ECPR as a time-limited intervention when obtaining advance preferences and during family meetings [45,46]. Given these predictable inflection points—particularly in ECPR—early palliative care involvement is not an “add-on,” but a core Phase I reliability support for communication, symptom preparedness, and family alignment.

## 5. The Role of Palliative Care Consultation

Elements of a “good death” include effective symptom relief, decisional clarity, preparedness, completion, contribution, and affirmation of personhood [47]. Palliative care consultation (PCC) in ECMO discontinuation supports patients and families through assessment of illness understanding, provision of emotional and spiritual support, facilitation of patient-centered goals-of-care discussions, and delivery of comfort-focused care at the end of life. Early involvement allows families—who often have limited familiarity with ECMO—to be prepared for the possibility of non-recovery and the need for discontinuation, while providing anticipatory guidance and aligning expectations. Palliative care clinicians also play a critical supportive role. They longitudinally assess coping and symptom distress and build rapport through repeated encounters. Building rapport is particularly effective when a small, consistent team follows the patient throughout the hospitalization and explores patient and family understanding, expectations, hopes, and fears. In addition, palliative teams assist with symptom-management planning during ECMO withdrawal, emphasizing pre-emptive comfort measures given the risk of rapid physiological deterioration after discontinuation [48,49]. The palliative care team functions as both a clinical resource and a process stabilizer, helping teams translate agreed goals into a coordinated, comfort-first plan.

In patients receiving ECMO, PCC utilization varies widely, with reported rates ranging from approximately 19% to 81% [50,51]. Institutions without embedded or automated palliative care triggers in the electronic health record tend to have lower rates of PCC utilization. Our institutional ECMO protocols have an automated consult feature to initiate PCC. During the beginning of the ECMO run, palliative care involvement is tailored to the patient and family needs; however, if CED is needed, the palliative care team always meets with the patient and/or family. We also have a separate staff wellness program available at the institutional level for staff involved in the case who choose to avail themselves of the wellness program. In a multicenter observational study, Dillon et al. found that patients who received PCC prior to ECMO cannulation had shorter durations of extracorporeal life support compared with those who received consultation later during their ECMO course, and PCC was also associated with a higher likelihood of transition to do-not-resuscitate status, suggesting earlier alignment of goals of care rather than a direct causal effect on outcomes [51]. In a separate cohort of patients with COVID-19–associated ARDS requiring ECMO, Rao et al. demonstrated that automatic PCC supported identification of surrogate decision makers, provided psychosocial support, and assisted with symptom management, thereby reducing clinician burden and improving care processes during critical illness [52]. These benefits depend on trust and communication quality. Phase I also explicitly addresses relationship-building and shared decision-making as operational prerequisites for a humane and reliable withdrawal process.

### Building Trust and Optimizing Shared Decision-Making

Qualitative studies of life support withdrawal in intensive care more broadly suggest that family involvement in withdrawal of life-sustaining treatment is frequently experienced as emotionally overwhelming and distressing, with some families describing the process as traumatic [49]. Trust in the treating physician plays a critical role, as families are less likely to agree to withdrawal when trust in the clinician–family relationship is lacking [50]. In ECMO, where technology intensity is high and prognostic uncertainty may be prolonged, deliberate trust-building and transparent expectation-setting become even more consequential for alignment. REMAP provides an evidence-informed structure for these conversations, explicitly integrating re-framing, emotion handling, values mapping, and recommendation-making—tasks that are central to Phase I alignment [18,19,20].

Family satisfaction with end-of-life care in the ICU is strongly influenced by how the transition off life-sustaining treatment is conducted and communicated. Satisfaction increases when withdrawal occurs over a deliberate time course rather than abruptly, when families feel heard rather than lectured during family conferences, and when clinicians explicitly acknowledge the emotional difficulty of having a loved one in the ICU, making decisions, and witnessing death. Families consistently value reassurance that pain and suffering will be actively treated, that their loved one will not be abandoned after withdrawal of life-sustaining interventions, and that their decisions will be respected. Together, these findings underscore the importance of empathic communication, anticipatory guidance, and visible commitment to comfort during transitions to end-of-life care, and are supported by structured approaches such as REMAP [18,19,53,54,55,56]. These communication practices are therefore not merely “soft skills,” but core Phase I safeguards that reduce predictable failure modes (mixed messaging, delayed symptom control, and unanticipated physiologic changes) and support a comfort-focused bedside course that is consistent.

## 6. Discussion

The evidence base for CED remains limited. Most published guidance derives from practical experience, expert consensus, and single-center observational work, with very few comparative studies documenting or evaluating how withdrawal is operationalized at the bedside [7,27,28]. Quantitative data on symptom trajectories, optimal medication regimens, and family-reported outcomes during ECMO discontinuation are scarce. This limitation is especially consequential because CED is a low-frequency, high-stakes process in which preventable distress can arise from variation in preparation, medication timing, and communication choreography rather than from the goals-of-care decision itself.

Future work should develop patient- and family-centered outcome measures for end-of-life care on ECMO, adapt and validate existing ICU end-of-life and family-experience measures for ECMO contexts (e.g., QODD and FS-ICU), and pair these with family psychological outcomes (PICS-F) and bereavement endpoints [15,31,57], while also testing structured CED bundles using pragmatic designs (e.g., stepped-wedge cluster randomized trials) [58]. Candidate “mechanism” studies should quantify the impact of pre-emptive medication timing (particularly in VA ECMO) and evaluate protocolized approaches to ventilator changes and sweep gas/blood flow reduction on observed distress and clinician-rated comfort. Qualitative studies should explore family experience and staff moral distress to refine scripts, anticipatory guidance, and debriefing/defusing formats, recognizing that the highest-yield refinements may be operational (who says what, when, and how) rather than pharmacologic alone [30,32].

Implementation science can clarify how community programs adopt CED reliably. Pragmatic registries could add CED process variables to existing ECMO datasets to accelerate learning and enable benchmarking on reliability measures (e.g., pre-brief completion, medication-before-ECMO-change, documented anticipatory guidance) rather than relying solely on downstream endpoints.

Institutions should track a small set of low-burden, high-signal process measures to guide improvement. Using a standardized brief, non-punitive data form and reviewing data quarterly will accelerate program learning and support run-chart feedback to teams. We recommend including the following: pre-brief performed (Y/N); palliative care involvement (Y/N); comfort medication before flow change (Y/N); extubation attempted (Y/N/NA); alarms silenced (Y/N); debrief completed within 7 days (Y/N); family feedback collected (Y/N); free-text fields for what went well and what needs improvement. Where feasible, add timestamps for (i) first comfort bolus/infusion start, (ii) first ECMO parameter change, and (iii) time of extubation (if performed), because medication-to-device timing is a plausible, modifiable driver of distress. Balancing measures, such as medication-related adverse effects, staff distress, and increased staff workload, should also be included to assess for unintended consequences of implementing a CED protocol.

Simulation of ECMO discontinuation with pumps/task trainers builds team skill in roles, alarm management, and communication choreography, and can be leveraged to rehearse the “comfort-first” sequence before sweep/flow reduction and extubation [59,60]. Given the moral and emotional load of CED, we recommend embedding palliative care in training sessions and pairing simulation with brief, structured post-event debriefing to reduce moral residue and capture system fixes (medication access, room set-up, staffing). A recent UK national consensus identified key research priorities, including the development of structured frameworks for ECMO withdrawal, communication tools to better support families, and quality improvement strategies to reduce variability and distress during this high-risk transition [7].

This review provides an expert-informed approach to CED. The checklist and four-phase model will require adaptation to local institutions. There are many different ECMO devices that will each have a unique protocol for silencing alarms and weaning. ECMO centers also have different staffing models, and it is important to note that when two different staff members are weaning the ECMO support and providing medications to the patient, there is a notable increase in the amount of care coordination required to provide the patient with smooth ECMO discontinuation. The overview protocol we provide here can serve as a template for ECMO centers to align with their local protocols, devices, and specialists (Table 1).

## 7. Conclusions

CED requires ethical clarity, anticipatory communication, thoughtful symptom management, and deliberate technical execution. By applying shared decision-making, aligning expectations early, and using a phased approach (family meetings, clinical preparation, tailored technical discontinuation, and bereavement/team support), ICU and ECMO teams can deliver dignified, goal-concordant EOL care. Framing CED as a low-frequency, high-stakes clinical process—not solely an ethical decision—highlights that preventable suffering and family trauma often arise from avoidable process variability (medication timing, role clarity, environmental control, and communication choreography). Checklists, role scripts, and debriefing practices can improve reliability and experiences for families and clinicians, while also creating a learning system that standardizes best practice, supports clinicians, and accelerates evidence generation for this vulnerable ICU moment.

## Figures and Tables

**Table 1:** Checklist for Compassionate ECMO Discontinuation.

Phase I	DOMAIN 1: Ethical Alignment and Shared Understanding
	Confirm palliative care involvement for family and staff support. Elicit and document patient values and family priorities, including preferred location of death and who should be present. Verify that interdisciplinary consensus is documented that discontinuation of ECMO is consistent with the patient’s goals of care and current clinical trajectory. Confirm that goals-of-care discussions have occurred with the patient (if capable) and/or appropriate surrogate decision-makers. Ensure code status is aligned with comfort-focused goals prior to withdrawal. Confirm all involved teams (ICU, ECMO, nursing, RT, perfusion, and palliative care) share a common understanding of the plan. Confirm contingency plan if unexpected circuit failure occurs prior to CED.
Phase II	DOMAIN 2: Family Communication and Preparation
	Review the planned sequence of withdrawal and anticipated physiologic changes. Confirm family understanding of the dying process, including modality-specific differences. Verify that the anticipated timing of death has been discussed, emphasizing variability (minutes to hours; occasionally longer). Assess and support spiritual, cultural, and religious needs. Offer and facilitate rituals, memory-making, and legacy needs (music, prayer, keepsakes). Review how unplanned circuit failure will be managed if it occurs prior to CED.
	DOMAIN 3: Team Preparation, Coordination, and Process Breakdown
	Pre-Withdrawal Safety Time-Out (REQUIRED) Clarify roles for bedside RN, RT, ECMO specialist/perfusionist, and primary team. Verify symptom management plan and medication availability. Verify ventilator management plan. Verify plan for discontinuation of adjunctive life-sustaining therapies (vasoactive medications, CRRT, mechanical circulatory support). Review planned device and therapy deactivation sequence.
	DOMAIN 4: Preventing Unrecognized or Undertreated Suffering
	Anticipate risks of dyspnea, air hunger, agitation, pain, and anxiety during CED. Acknowledge ECMO-related pharmacokinetic considerations (circuit sequestration, tolerance, rapid physiologic shifts) that may necessitate higher-than-usual medication dosing if initiating new medications to support the comfort transition. Utilize institutional ICU comfort care order set or similar orders. Verify that neuromuscular blockade has been discontinued. Confirm neurologic responsiveness (e.g., 4/4 twitches on train-of-four) prior to assessing comfort and titrating medications. Confirm comfort-focused medications (opioids, benzodiazepines, etc.) are at bedside. Prepare adjunct comfort measures (anticholinergics for secretions; non-pharmacologic supports such as quiet environment, fan, music, family presence). Ensure medications are administered before ECMO flow reduction, particularly for VA ECMO, to ensure systemic distribution.
Phase III	DOMAIN 5: Environmental Preparation—Preventing Chaotic Withdrawal
	Silence non-essential alarms; maintain essential monitoring (preferably remotely). Remove non-essential equipment from the room when feasible. Ensure spiritual support is present if desired by family.
	DOMAIN 6: Withdrawal of Adjunctive Therapies
	Return blood from CRRT circuit and remove CRRT from the room. Manage secretions prior to extubation. Deactivate ICD/anti-tachycardia therapies (magnet only as temporary bridge). If extubation is desired, confirm comfort on minimal ventilator support prior to extubation. Discontinue vasopressor and inotrope infusions. Anticipate and prepare for acute pulmonary edema or abrupt dyspnea/increased work of breathing, especially in patients previously requiring high ventilator support.
	DOMAIN 7: ECMO Specific Discontinuation
	VV ECMO Specific Recommendations: Adjust alarms to silent per device-specific protocol. Set low-flow alarm to 0 L/min. Recommend weaning blood flow to 2–3 L/min. Recommend maintaining FdO_2_ at 100% and focusing on sweep gas flow reduction. Titrate comfort medications continuously to prevent dyspnea or distress. Reduce sweep gas to zero by titrating down every 5–10 min, allowing time for additional medication administration in between titrations. VA ECMO Specific Recommendations: Address concurrent mechanical circulatory support (balloon pump or ventricular assist device) by weaning and then deactivating the device per device protocol. Set low-flow alarm to 0 L/min. Recommend maintaining FdO_2_ at 100% and sweep gas flow at current level to focus on blood flow reduction. Wean blood flow/pump speed to the lowest level per device-specific protocol (Cardiohelp, Centrimag, VitalFlow, LifeSPARC, etc.). Titrate comfort medications continuously to prevent dyspnea or distress. Ensure the last bolus is given before blood flow is 0 L/min to allow medication circulation. Clamp circuit and power off the device. Cover the ECMO circuit to reduce visual distress as blood separates within tubing.
Phase IV	DOMAIN 8: Documentation—Preventing Ambiguity and Moral Residue
	Document clearly and consistently using neutral, non-stigmatizing language (e.g., “compassionate ECMO discontinuation with death expected”). Record sequence of withdrawal, symptom management strategies, and family presence.
	DOMAIN 9: Aftercare & Support—Preventing Complicated Grief and Moral Injury
	Ensure privacy and unhurried time for family rituals and goodbyes after death. Provide bereavement resources. Capture learning and system-level issues (alarms, medication availability, room setup, staffing) for quality improvement before future CED events. Perform a team debrief (structured, non-punitive) for clinicians, ECMO specialists, nurses, RTs, and other involved staff.

## Data Availability

No new data were created or analyzed in this study. Data sharing is not applicable to this article.

## Supplementary Materials

The following supporting information can be downloaded at: https://www.mdpi.com/article/10.3390/healthcare14091249/s1, S1: PubMed Search Strategy; Table S1: Classification of Evidence Sources Included in Narrative Review.

## Appendix A

### Shared Decision-Making Scripts for Compassionate ECMO Discontinuation (CED)

* Appendix A, Appendix B were developed by the authors, informed by REMAP and the Ariadne Labs Serious Illness Conversation Guide; wording has been adapted and ECMO-specific content added. Any errors are the authors’ [18,19,20].

1. Set the stage

“Thank you for meeting with us. I know this has been an incredibly long and heavy journey. We want to talk together about where things stand now and make sure our plan reflects what matters most to [patient’s name].”

2. Assess understanding

“Before I explain our concerns, can you tell me what you understand about [patient’s name]’s condition and what the ECMO is doing for them right now?”

Listen, affirm and correct gently.

3. Reframe and Explain

“ECMO is a life-support machine that has been doing the work of [heart/lungs] for X time. However, something has changed. ECMO is no longer helping [patient’s name] move toward recovery. Despite everything we’ve tried, there are no remaining treatments that can help [patient’s name] recover enough to come off ECMO or reach another goal like transplant or long-term recovery. At this point, ECMO is no longer helping them get better—it is only keeping their body going.”

4. Name the decision explicitly

“Because of that, we are at a point where we need to decide whether continuing ECMO still matches [patient’s name]’s values and goals, or whether the more compassionate option is to focus entirely on comfort and allow a natural death by discontinuing ECMO.”

(Pause for emotion. Acknowledge.)

5. Elicit values and goals

“When [patient’s name] was at their best, what mattered most to them in life?”

“Did they ever talk about what they would find unacceptable — things they would not want to live with?”

“If [patient’s name] could see this situation now, what do you think they would say is most important?”

6. Align recommendation with values

“Based on what you’ve told me—that [summarize values]—continuing ECMO would not help them reach those goals.”

7. Make a recommendation

“For that reason, our medical recommendation is to discontinue ECMO and focus fully on comfort, dignity, and being surrounded by the people they love.”

8. Describe what compassionate ECMO discontinuation looks like

“If we move forward with this, our priority is comfort. We would give medications to make sure there is no pain, air hunger, or psychological and physical distress. We would prepare the room so it is quiet and private. You can choose who is present, whether there is music or prayer, and how involved you want to be.”

9. Check readiness and invite questions

“How does this plan sit with you right now?”

10. Confirm next steps

“We don’t have to do this today unless you feel ready. If you’d like, we can take time, involve chaplaincy or other family members, and answer more questions. When you are ready, we will plan this carefully and walk with you through every step.”

## Appendix B

### Communication Toolkit: Long-Form Scripts

Appendix A, Appendix B were developed by the authors, informed by REMAP and the Ariadne Labs Serious Illness Conversation Guide; wording has been adapted and ECMO-specific content added. Any errors are the authors’ [18,19,20,61]

Communication Toolkit: Long-Form Scripts	
Opening (Ask–Tell–Ask)	“Could I share what we are seeing and hear what matters most to you? Given how important comfort and togetherness are to you, we recommend a plan that focuses on keeping [patient] comfortable while we say goodbye. That plan involves turning off the machine that has been doing the work of the heart/lungs, which means [patient] will die peacefully here with you.”
Explaining Technicalities	“For this type of support, when we stop the machine, circulation will fall within minutes. You may see the monitor numbers change and [patient] become less responsive. We give medication first so [patient] does not feel air hunger or distress.”
Language When There Is Disagreement	“I hear how strongly you are hoping for recovery—we wish for that as well. At the same time, we want to avoid treatments that prolong suffering without changing the outcome. One option is a clearly defined time-limited trial: if we do not see evidence of *[specific recovery marker]* by *[time]*, and if transplant or durable support is not feasible, we would then shift entirely to comfort and discontinue ECMO.”
Narrating the Sequence	“We’re starting medications now to ensure comfort. We’ll reduce the ECMO support gradually and pause at each step to reassess comfort. We’ll stay with you throughout and explain what’s happening as we go.”

## Appendix C

### Compassionate ECMO Discontinuation (CED)—ICU Procedure Note

**Patient/Identifiers:** Name: ______ Medical Record Number: ______ Location: ______**Primary diagnosis/indication for ECMO:** ______ **ECMO day #:** ______**Date/Time:** ______ (start of withdrawal sequence)**ECMO Type:** _ VA _VV _VPA _Alternate strategy:______ **(cannulation sites: ______; most recent blood flow: ______ L/min; sweep gas flow: ______; FdO_2_: ______)**

**Phase I—Anticipation & Alignment**

1) **Clinical Rationale & Prognosis:**

a) Prognosis reviewed in plain language, including expected outcomes with and without ongoing ECMO. Despite maximal supportive therapy, the likelihood of meaningful recovery is poor, and continued ECMO is no longer consistent with the patient’s goals or expected clinical trajectory.

2) **Shared Decision-Making:**

a) Discussion held with the patient (if decisionally capable) and/or legally authorized surrogate(s). Capacity assessed and documented as: ______. Participants included ICU team, ECMO team, and palliative care (as available). Interpreter used: _No _Yes (language: ______). Patient values/goals were reviewed, with emphasis on comfort, dignity, and family presence.

3) **Plan & Code Status:**

a) Multidisciplinary recommendation for compassionate discontinuation of ECMO was reviewed and agreed upon. Code status updated/confirmed as consistent with comfort-focused care (e.g., DNR/DNI/Comfort Measures Only), with no escalation of life-sustaining therapies.

**Phase II—Preparation**

4) **Withdrawal preparation:**

a) Comfort-focused withdrawal plan established with sequencing of device and therapy changes. A brief bedside time-out was performed (roles confirmed; family updates planned; alarms/lines prepared). Anticoagulation plan addressed: ______. Comfort medications were administered *before* any reduction in ECMO support and were titrated throughout to maintain comfort (agent(s)/doses documented in MAR). Additional life-sustaining therapies addressed (ventilator/vasopressors/CRRT) with a comfort-first approach: ______.

5) **Notifications & support services:**

a) Relevant consulting services notified of CED timing as appropriate. Spiritual care, social work, and interpreter services offered, with attention to cultural and religious needs. Per institutional policy, required donation/referral notifications were completed as applicable: ______.

**Phase III—Implementation**

6) **CED Implementation Summary (document sequence):**

a) **Pre-medication given at:** ______; **comfort reassessed at:** ______
b) **ECMO adjustments** (e.g., gradual reduction vs direct cessation; sweep/flow changes; clamp/stop): ______
c) **Cannula management after cessation** (left in place vs removed; hemostasis plan): ______
d) **Ventilator/oxygen strategy during/after cessation** (including extubation if performed): ______

7) **Team presence:**

a) Appropriate interdisciplinary team members present during CED (ICU attending/fellow/APP: ______; ECMO specialist/perfusion: ______; bedside RN: ______; RT: ______; palliative care: ______; chaplain/social work: ______).

8) **Family support:**

a) Family presence and privacy supported. Anticipatory guidance provided regarding expected physiologic changes and what the family may observe.

**Phase IV—Aftercare & Learning Capture**

9) **Death Pronouncement:**

a) **Time of death:** ______
b) **Pronouncing clinician:** __________________
c) **Family present at time of death:** _No _Yes (names/relationship: ______)
d) **Exam/criteria documented (as applicable):** absence of pulses/heart sounds/respirations; fixed pupils; asystole on monitor: ______

10) **Family Aftercare**

a) Bereavement resources offered. Any requested rituals honored where feasible.

11) Team Aftercare & Learning

a) Post-CED debrief held or scheduled

**Attending signature:** __________________ **Date/Time:** ______

## Appendix D

Care Team Roles [7,8]

Care Team Roles	
ECMO Attending Intensivist	Leads clinical decision making, frames goals with the family, assigns roles, and oversees the sequence. Ensures documentation and communication with consulting teams.
ECMO RN Specialist/Perfusionist	Plans the technical sequence, prepares clamps, caps, and drapes, silences console alarms, and executes clamp-and-stop or clamp–cut steps.
Bedside Nurse	Prepares and administers medications, monitors comfort, manages lines and devices, and assists with environment setup and post-mortem care.
Respiratory Therapist	Optimizes ventilator settings for comfort, manages secretions, supports extubation if chosen, and coordinates alarm silencing.
Palliative Care Clinician	Guides communication, provides symptom-management expertise, supports family needs, and co-leads debriefs.
Spiritual Care Team Member/Chaplain	Facilitates spiritual, religious, or cultural rituals, provides bedside presence to support families

## Appendix E

### Example Local CED Operational Protocol

Appendix E was developed by the authors and is our protocol based on the equipment and order sets available our home institution.

**ECMO Transition to Comfort Focused Care – Adult ECMO Clinical Practice Guideline**

**PURPOSE:**

The purpose of this guideline is to provide a standardized approach to the withdrawal of life sustaining ECMO support if survival is no longer thought possible and the transition to comfort focused care for patients on VA (Veno-Arterial) and VV (Veno-Venous) ECMO.

**DEFINITIONS:**

1. ECMO (Extracorporeal Membrane Oxygenation): The use of a modified cardiopulmonary bypass circuit for temporary life support for patients with potentially reversible cardiac and/or respiratory failure by providing a mechanism for gas exchange and/or cardiac support.
2. VA ECMO (Venoarterial Extracorporeal Membrane Oxygenation): Provides hemodynamic support in addition to respiratory gas exchange. Venous blood is drained, passes through a centrifugal pump and membrane lung (where O2 is added and CO2 is removed), then returned into an artery. During VA ECMO, blood within the circuit bypasses the native heart and lungs.
3. VV ECMO (Venovenous Extracorporeal Membrane Oxygenation): Provides respiratory gas exchange. Venous blood is drained, passes through a centrifugal pump and membrane lung (where O2 is added and CO2 is removed), then returned into an artery. During VV ECMO, blood within the circuit does not bypass the native heart and lungs.
4. ECMO Intensivist: ECMO trained critical care physician.
5. ECMO Specialist: ECMO trained critical care nurse.
6. ECMO CPG: ECMO Clinical Practice Guideline
7. F_s_O_2_: Fraction of oxygen delivered by sweep gas
8. Sweep gas: Oxygen and medical air blend delivered to the oxygenator, measured in liters per minute (LPM), responsible for decarboxylation (CO_2_ removal)
9. Blender: Sweep gas delivery device through which F_s_O_2_ and LPM can be controlled independently
10. Blood flow: Blood flow through the ECMO circuit as determined by setting of RPMs (revolutions per minute)
11. Impella: Percutaneous ventricular assist device. May be used in ECMO to offload or decompress the left ventricle.
12. IABP: Intra-aortic balloon pump. May be used in ECMO to offload or decompress the left ventricle.

**GUIDELINE DETAILS:**

*General:*

1. Use the ICU Comfort Care Order Set—*ICU: COMFORT CARE: WITHDRAWAL OF MECHANICAL VENTILATION (PO-7136)*

a. Medications for comfort
b. If indicated, paralytic cessation with train of four monitoring
c. Extubation if desired by family (no SBT is required)
d. Order to wean and stop ECMO circuit at direction of ECMO attending

2. *This is a very difficult decision, ensure that palliative care is directly involved for family and staff support*
3. If the patient is on neuromuscular blockade, this infusion must be stopped prior to transitioning to comfort focused care. Ensure 4/4 twitches on train of four testing.
4. Ensure comfort focused medications (opiates, benzodiazepines, antipsychotics) are available.

a. Opiate infusion
b. Benzodiazepine as either push dose or infusion
c. Haldol as needed for terminal agitation/restlessness
d. Glycopyrrolate for secretion management

5. If the family desires extubation as part of the comfort care process, reduce ventilator support to PSV 5/5/0.4 while titrating comfort medications. Once the patient is comfortable on minimal settings, proceed with extubation.
6. Turn off vasopressor and inotrope infusions.

*VV ECMO Specific*

1. Maintain the F_S_O2 at 100%
2. Wean the blood flow to 2.0–3.0 L/minute
3. Begin weaning the sweep gas as follows until you reach sweep of 0:

a. If sweep > 5 then wean by 2 every 5–10 min titrating medications as needed to maintain patient comfort and reduce air hunger
b. If sweep < 5 then wean by 1 every 5–10 min titrating medications as needed to maintain patient comfort and reduce air hunger
c. Some patients may require smaller titration of sweep between 1 LPM and 0 to allow for symptom management.

4. Once sweep is at 0, continue comfort focused medications until patient passes. It is not necessary to clamp the circuit because blood flow can continue. If blood flow is stopped, or RPMs dropped under 1500, the circuit should be clamped to avoid retrograde flow and negative flow alarms.

*VA ECMO Specific*

1. If there is an Impella, walk the P-level down to P0. Wean the Impella to P0 by 1 P level every 5–10 mins. Titrate medications as needed for patient comfort. Once at P0, unplug the driveline from the console and turn the Impella off.
2. If there is an IABP (Intra-Aortic Balloon Pump), wean the balloon pump to 1:3 over a 10 min period and then turn the balloon pump off. Titrate medications as needed for patient comfort.
3. Maintain F_S_O2 at 100% and sweep gas flow rate at current level
4. Set ECMO low flow alarm at 0 L/min.

a. If on Cardiohelp device put in global override mode. Wean pump speed to 1500 rpm then clamp circuit. Turn device off. Titrate medications as needed for patient comfort throughout.
b. If on Centrimag device—wean pump to 1500 RPMs, clamp circuit, manually reduce pump speed to 0. Turn device off. Titrate medications as needed for patient comfort throughout.
c. Note that unplugging the machine will result in loud beeping, even after device is turned off.

5. Cover the ECMO circuit with a bedsheet (the blood will separate in the tubing which may be disconcerting to family).
