# Supplementary material for: Compassionate Extracorporeal Membrane Oxygenation Discontinuation: A Narrative Review and Practical Process Model for Reliable End-of-Life Care
Source: Healthcare (Basel). 2026 May 6;14(9):1249. doi: 10.3390/healthcare14091249 (PMC13163547; doi:10.3390/healthcare14091249)
Supplement: Supplementary file 1 [file healthcare-14-01249-s001.zip › healthcare-4108983-supplementary.pdf]

Supplementary Material S1

PubMed Search Strategy

https://pubmed.ncbi.nlm.nih.gov/?term=(%22Extracorporeal+Membrane+Oxygenation%22[MeSH+Terms])+AND+(%22Withholding+Treatment%22[MeSH+Terms]+OR+%22Palliative+Care%22[MeSH+Terms]+OR+%22Terminal+Care%22[MeSH+Terms]+OR+%22Life+Support+Care%22[MeSH+Terms]+OR+%22Ethics,+Medical%22[MeSH+Terms])+AND+english[Language]+AND+(0001/01/01:2025/10/19[dp])

Table S1: Classification of Evidence Sources Included in Narrative Review

**Evidence Category**	**Description**	**Examples in This Review**	**Role in Framework**
Empirical Studies	Observational cohorts, pharmacokinetic studies	PK/PD ECMO sedation studies; palliative consult outcomes	Inform symptom management and process risks
Consensus Guidelines	Professional society statements	ELSO, SCCM, UK VV-ECMO withdrawal consensus	Define best-practice structure
Ethical Analyses	Normative frameworks, policy papers	AMA ethics, ATS policy statements	Guide decision-making boundaries
ICU EOL Literature	Non-ECMO withdrawal studies	FS-ICU, QODD, withdrawal communication studies	Provide transferable process insights
Expert/Institutional Practice	Protocols and experiential models	Institutional CED workflow (Appendix 3)	Operationalize bedside execution
